# Immunogenetics and pharmacogenetics of allergic asthma in Africa

**DOI:** 10.3389/falgy.2023.1165311

**Published:** 2023-05-09

**Authors:** Tshegofatso Mabelane, Refiloe Masekela, Collet Dandara, Sabelo Hadebe

**Affiliations:** ^1^ Department of Medicine, Sefako Makgatho Health Science University, Ga-Rankuwa, South Africa; ^2^ Department of Paediatrics, Nelson Mandela School of Medicine, Inkosi Albert Luthuli Hospital, University of KwaZulu-Natal, Durban, South Africa; ^3^ Division of Human Genetics, Department of Pathology, Faculty of Health Sciences and Institute of Infectious Diseases Molecular Medicine, University of Cape Town, Cape Town, South Africa; ^4^ Platform for Pharmacogenomics Research and Translation, South African Medical Research Council, Cape Town, South Africa; ^5^ Division of Immunology, Department of Pathology, Faculty of Health Sciences, University of Cape Town, Cape Town, South Africa

**Keywords:** asthma, Africa, pharmacogenetics, genetics, th2 (type-2) immune responses

## Abstract

Asthma is a common chronic condition in children and in an African setting is often highly prevalent in urban areas as compared to rural areas. Asthma is a heritable disease and the genetic risk is often exacerbated by unique localised environmental factors. The Global Initiative for Asthma (GINA) recommendation for the control of asthma includes inhaled corticosteroids (ICS) alone or together with short-acting β_2_-agonists (SABA) or long-acting β_2_-agonists (LABA). While these drugs can relieve asthma symptoms, there is evidence of reduced efficacy in people of African ancestry. Whether this is due to immunogenetics, genomic variability in drug metabolising genes (pharmacogenetics) or genetics of asthma-related traits is not well defined. Pharmacogenetic evidence of first-line asthma drugs in people of African ancestry is lacking and is further compounded by the lack of representative genetic association studies in the continent. In this review, we will discuss the paucity of data related to the pharmacogenetics of asthma drugs in people of African ancestry, mainly drawing from African American data. We will further discuss how this gap can be bridged to improve asthma health outcomes in Africa.

## Introduction

Asthma is one of the most common non-communicable diseases, affecting an estimated 262 million people in 2019 (1). An International Study of Asthma and Allergies in Childhood (ISAAC) has provided the most reliable global, comparative data on the prevalence of asthma and other allergic conditions in children, enabling comparison of asthma prevalence between different parts of the world (2). In this study, the prevalence of asthma in 13–14-year-old Black African children was 15.3%, which was higher than the global average (14.1%) (3). In this study, the prevalence of asthma in Africa was determined among 16 countries and 22 centres (3). Several centres, including Cape Town, South Africa (SA) (20.3%), Polokwane, SA (18.0%), Reunion Island (21.5%), Brazzaville, Republic of Congo (19.9%), Nairobi, Kenya (18.0%), Urban Ivory Coast (19.3%) and Conakry, Guinea (18.6%) showed relatively high asthma symptom prevalence similar to those in western Europe (3). These findings are analogous to previous studies conducted in Africa, depicting the prevalence of asthma to be higher in urban than rural areas (3–6). A few recent studies conducted in the Western Cape and Eastern Cape in South Africa comparing rural and urban children showed a significant impact of geographical location in the development of allergic disease in children under the age of 12 years (7, 8). Atopic dermatitis is much lower in rural areas compared to urban areas which have a prevalence similar to western countries (9). Environmental bacterial endotoxin have a strong protective correlation, where high levels are observed in rural areas and less so in urban areas. Interestingly, levels of house dust mites are comparable between urban and rural areas (9). These trends are similar to those that have been shown elsewhere (10, 11). ISAAC provided the prevalence of asthma in Africa, however, the underlying factors responsible for genetic determinants of asthma still need to be investigated.

While the risk factors for asthma are well known and managed in high resource settings, such healthcare systems do not exist in some parts of Africa. This leads to poor and late diagnoses of asthma in children and adults, many deaths go unreported and prevalence may be higher than current estimates (12, 13). The burden of other infectious diseases in Africa means that generally noncommunicable diseases including asthma are largely neglected (14). The increased number of people dying due to asthma is alarming when the disease itself is treatable in most cases by medication available in public hospitals (15, 16). Inhaled corticosteroids (ICS) together with inhaled short-acting β_2_-agonists (SABA) are the mainstay for treatment as they can act as a short reliever. However, the lack of simple inhaler devices that can reliably deliver a known drug concentration leads to reduced treatment efficacy in low and middle-income countries (LMICs) (15). Affordability and availability of first-line asthma drugs or their generic equivalents in public hospitals contribute to poor control of asthma (17). Local solutions such as homemade spacers from 500 ml plastic bottles have been shown to be effective for children with asthma (18). Part of the problem lies in that most people don't recognise symptoms and have never been diagnosed to have asthma. In cases where people have been properly diagnosed, with limited educational support, they may poorly adhere to prescribed medication. Again, due to the complex nature of the disease, patients coming to hospitals are likely to have acute symptoms and often drugs for the management of acute asthma, oxygen, oral corticosteroids, and spacer devices are not included in the World Health Organization's (WHO) essential medicines list and likely not to be available in local hospitals.

## Asthma risk factors

The major risk factors for the development of asthma include genetic variation and environment. Research supporting the role of environmental risk factors for the development of asthma includes nutrition (1), obesity (19), antibiotic use during pregnancy and in infants as well as toddlers (20) and psychosocial factors (21, 22). Other risk factors that are key in Africa are the presence of eczema (23), allergen exposure [e.g., pet (24), house dust mite, cockroach, mould (25)], industrial pollution (26) and informal housing (24). The genetics of asthma can be described at three levels, (i) genetic variability that differentiates those who are more likely to develop asthma, immunogenetics, (ii) genomic variability that affects or determines how asthma patients respond to a given medication, pharmacogenomics, (iii) the genetics of asthma-related traits (i.e., lung function, asthma exacerbations, IgE levels, asthma severity). The association between genetics and asthma is dissected separately in this article.

## Immunogenetics of asthma

Twin studies have suggested that between 60% and 80% of asthma disease is heritable, meaning genetics are crucial in outcomes (27–32). Genetic-wide association studies (GWAS) have identified variations in genes associated with the immune system to be critical in asthma development (33–35). These genetic risk alleles span across the genome and are found in chromosome 2 (IL-1R1, rs1558641), chromosome 5 (RAD50, rs6871536), chromosome 7 (CDRH3, rs6967330), chromosome 9 (IL-33, rs928413) and chromosome 17 (GSDMB, rs2305480) (33). It is evident that there is multiple genetics involved in the immunopathology of asthma, thus studies have dissected susceptible genes in different ethnic groups. There is a measurable difference in asthma phenotypes across ethnicities (36, 37). Although some of these differences can be attributed to healthcare affordability, some are due to inherited genetic variation. Studies with admixed populations have suggested that genetic variation determines asthma severity (38). African ancestry impacts lung function and people of African ancestry show low baseline lung function when compared to people of European ancestry (39). Adult people of African ancestry show increased genetically associated asthma exacerbations independent of treatment management and other socio-economic factors (40). Interestingly, in children of African ancestry, both genetic and socioeconomic factors influence hospital re-admission rates for uncontrolled disease when compared to European-ancestry children admitted for the same symptoms (41). These underlying genetic variants within specific populations have implications for early disease diagnosis, misclassification and response to treatment. Asthma-related morbidity and mortality are higher among Puerto Rican and African Americans compared to non-Hispanic white people and Mexican Americans (42).

Some of the more prominent genetic variations are in the 17q12-21 locus harbouring genes such as GRB7, IKZF3, ZPBP2, GSDMB, ORMDL3, GSDMA and the 6p21.32 locus harbouring HLA genes such as HLA-DQ and HLA-DR and appear to be the main asthma contributors identified through GWAS (32, 43). Of the one hundred and twenty-six asthma-associated independent variants (*p*-value <5.0 × 10^5^) identified to date, the majority belong to the 17q12-21 locus and have been replicated in multiple ancestries (44, 45). Signals near the HLA-DQ gene were found in European American, African American, and Latino populations; but with different SNPs identified in each ethnic group (31). Furthermore, class II HLA alleles appear to be involved in late-onset allergic asthma in European and Hispanic populations (46–48). Interestingly, the HLA-DRB1*09:01 allele was associated with increased total Immunoglobulin E levels in African ancestry individuals with asthma, potentially through a mechanism involving specific peptide presentation and/or increasing inflammation (49). More recently, a consortium on Asthma among African Ancestry Populations (CAAPA) has identified two novel loci, 8p23 (associated with ARHGEF10 or MYOM2 genes) and 8q24 (associated with intronic TATDN1) in chromosome 8 (50). These loci although not yet validated in an independent study are associated with asthma risk in the African ancestry population (50). Studies have evidently established that genetic associations for asthma vary amongst different racial and ethnic groups (31, 34, 50). While these studies in African American and other populations with African ancestry highlight ethnic-specific variants leading to asthma risk, it is very likely that as more GWAS studies are performed in Africa, they would be a local environmental influence on genetic risk factors. Although more than 100 SNPs have been identified to associate with asthma across multiethnic GWAS studies, only a small fraction explains asthma phenotype. This is largely influenced by the missing heritability problem, particularly in complex trait diseases like asthma, where environment, epigenetics and heritability overestimation can influence phenotype. Small effect size SNPs and technical limitations of GWAS imputations, especially when dealing with less represented genetic ancestries can also contribute to missing heritability (51, 52). Large meta-analysis GWAS studies that take into account the effect size and allele frequency across populations, and multi-ancestry imputation servers (e.g., CAAPA African American dbGAP (accession code phs001123.v1.p1, https://imputationserver.sph.umich.edu) would help in fine-mapping SNPs and reduce some of the missing heritability (52). Genetic heterogeneity in African ancestry is also caused by fewer genetic population bottlenecks and the fact that human evolution began in Africa. This translated to much lower linkage disequilibrium and heterogenous haplotypes due to less genetic recombination (53). All these factors need to be considered in multi-ancestry analysis of genetic variations in disease and more so in complex diseases such as asthma which are driven by common variants with small effect sizes.

## Management of asthma

The long-term goals of asthma management are to achieve good symptom control and to minimize future risk of asthma-related mortality, exacerbations, persistent airflow limitation and side effects of treatment (1). For individual patients, treatment decisions should take into account patient characteristics (phenotype) that predict the patient's likely response to treatment, together with the patient's goals or concerns and practical issues (inhaler technique, adherence, medication access and cost to the patient). Thus, effective asthma management requires a partnership between the patient (or the parent/carer) and their healthcare providers (1). Non-drug management of asthma includes environmental changes such as smoking cessation (especially exposure in children) and allergen avoidance (including occupational), weight reduction, avoiding drugs that are associated with triggering symptoms and managing comorbid diseases (e.g., allergic rhinitis, obesity, gastroesophageal reflux disease) (1). Overall, asthma treatment involves the stepwise approach whereby treatment is stepped up and down in response to asthma control and exacerbation risk (54).

Until recently, international asthma treatment approaches started with reliever medication (e.g., inhaled salbutamol) used “as required” at the lowest step with the addition of regular preventer medication, for example, ICS and other therapies including long-acting β_2_ agonists (LABA) at higher steps (54)^.^ Global Initiative for Asthma (GINA) guidelines recommend that the minimum treatment for asthma in adolescents and adults is either combined ICS-formoterol as reliever therapy (maintenance and reliever therapy) or ICS whenever SABA is taken (54). In children below the age of 6 years, LABA is contraindicated, hence ICS is used whenever SABA is as reliever therapy. Daily use of ICS is the mainstay of treatment in patients with troublesome asthma symptoms occurring at least more than twice a month, or waking due to asthma once a week or more, especially if any risk factors exist (1). The dose of ICS is increased in a stepwise manner, based on symptom control (55). Inhaled Corticosteroids work by binding to the intracellular glucocorticoid receptor (GR), which is encoded by the gene NR3C1 (56). Several proteins/ enzymes are then involved during the translocation of the ICs-GR complex to the nucleus where it binds to its cognate DNA sequences called glucocorticoid response elements (GREs) (57). The binding of the ICs-GR to GREs triggers the transcription of several genes. It is important to note that, the anti-inflammatory activity of ICs is achieved through a combination of inhibition and upregulation of gene transcription of different genes (58). Inhibition results in the reduction in the production of pro-inflammatory cytokines. On the other hand, transactivation results in an upregulated expression of annexin A1 which leads to reduced prostaglandin and leukotriene production. It is believed that transactivation plays a critical role in the onset of unwanted adverse events (ADEs) induced by ICSs (56, 57).

Furthermore, other preventer therapies such as LABA, and Leukotriene receptor antagonist (LRTA) may be added if patients do have complete control of symptoms (59). In severe symptoms and low lung function, long-acting anticholinergic (tiotropium) is added and a short course of oral corticosteroids (OCS) may be prescribed (60). Referral for phenotyping is recommended as a last step in the GINA guidelines, in order to add more targeted therapy such as biologics.

Management of acute asthma exacerbation includes nebulised drug administration initiated in the emergency department with the administration of oxygen aimed at achieving arterial oxygen saturation of 93%–95% in adults and adolescents and 94%–98% for children between 6 and 11 years. Inhaled SABA therapy should be administered frequently. The most cost-effective and efficient delivery is by pressurized metered-dose inhalers (pMDI) with a spacer (61, 62). Systemic corticosteroids speed the resolution of exacerbations and prevent relapse, and should be utilized in all but the mildest exacerbations in adults, adolescents and children 6–11 years (63, 64). The oral route of systemic corticosteroids is preferred because it is quicker, less invasive and less expensive (65), however, intravenous steroids may be administered in patients failing to tolerate them orally. Additional treatment (intravenous magnesium, high dose ICS) is indicated in severe patients with poor clinical response (1). For adults and children with moderate-severe exacerbations, treatment in the emergency department with both SABA and ipratropium, a short-acting anticholinergic, was associated with fewer hospitalizations and greater improvement in PEF and FEV1 compared with SABA alone (62, 63). However, ipratropium showed no benefit in children admitted for acute asthma.

## Pharmacogenomics of asthma

Differences in genetic ancestry might also be important in the therapeutic efficacy of commonly used asthma therapies. At least 50% of the variability in ICS response is thought to be due to individual characteristics of target genes (66). African Americans with moderate to severe asthma have reduced bronchoresponsivess compared to European ancestry individuals (67), while Puerto Ricans show less responsiveness to albuterol compared to other Hispanic groups (68). The most relevant is related to a commonly used class of pharmacologic agents: β_2_-adrenergic receptor agonists, which are divided into short-acting β-agonists (SABAs) and long-acting β-agonists (LABAs) (Table 1). SABAs include fenoterol, isoproterenol, pirbuterol, levalbuterol, and albuterol whereas LABA includes salmeterol and formoterol, which are often used in combination with inhaled corticosteroids for chronic management (55). SABAs are the most frequently prescribed asthma medication due to their ability to rapidly cause smooth muscle relaxation in the airways (69).

**Table 1:** Genes that determine the short and long β_2_-adrenergic receptor agonists response profiles in asthma.

Gene	Associated loci	Affected pathway or therapeutic drug
ADCY9	rs2230739	Inhaled β_2_-adregenrnic receptor agonists, SABAs
ADRB2	rs73650726, rs1042713	SABAs, albuterol
ARG2	rs2781667, rs7140310, rs10483801	SABAs, albuterol
SPATS2l	rs295137	LABAs, Salmeterol and formoterol
ADRB2	rs1042713, rs1800888	LABAs, Salmeterol and formoterol

SABA, short-acting β_2_-agonists; LABAs, long-acting β_2_-agonists.

## Inhaled corticosteroids

The importance of genetic variation in each of the genes in Table 2 derives from their normal function and the effects of disturbing that homeostasis. For example, genetic variation in NR3C1 causes modifications of the GR (56). The FKBP4 gene encodes for the immunophilin FKBP52, which regulates GR signalling (58). Corticosteroid metabolism occurs in the liver and most ICSs are metabolized to inactive metabolites. ICSs are substrates for CYP3A4, CY3A5 and transporters such as the multidrug-resistance gene MDR1 (ABCB1) (70). Mutations in any of these genes which are part of the pharmacokinetics and pharmacodynamics of ICSs contribute to variability in response observed among asthmatic patients. Importins (IPOs) are known to direct the ligand-activated GR to gated channels of the nuclear membrane and cause the translocation of the GR to the nucleus (56). Thus, genetic variation in IPO coding genes such as IPO13 is likely to affect the functions associated with its proteins (56).

**Table 2:** Genes that determine the genetic susceptibility and treatment corticosteroid response profiles in asthma.

Gene	Associated loci	Affected pathways, or therapeutic drugs
ABCB1 (MDR1)	rs1128503, rs2032582	Pharmacokinetics of corticosteroids
	rs1045642	
CRHR1	rs242941, rs1876828, rs242941	Corticosteroid pathway
CYP3A4	rs35599367 (3A4*22)	Pharmacokinetics of corticosteroids (e.g., fluticasone propionate)
CYP3A5	rs776746 (CYP3A5*3)	Pharmacokinetics of corticosteroids (e.g., beclomethasone)
DUSP1	rs881152, rs34507926	Corticosteroid pathway
FBXL7	rs10044254	Corticosteroid pathway
FCER2	(rs28364072, T2206C)	Modifies corticosteroid activity
FKBP4	rs4713916	Corticosteroid pathway
GLCCI1	rs37972	Modifies corticosteroid activity
HDAC1	rs1741981	Corticosteroid pathway
HDAC2	rs58677352	Corticosteroid pathway
IPO13		Pharmacokinetics of corticosteroids
miRNA	hsa-miR-142-3p; hsa-miR-17-5	hsa-miR-142-3p targets GC receptor- *α*
NR3C1	rs6189, rs6190, rs41423247, s56149945	Corticosteroid pathway
ORMDL3	rs2872507, rs72821893	Modifies corticosteroid activity
STIP1	rs6591838, rs223647, rs6591838, rs1011219	Corticosteroid pathway
TBX21	rs2240017	Modifies corticosteroid activity
RNASE2	rs3827907	ICS treatment response in the presence of eosinophilic inflammation
APOBEC3B and APOBEC3C	rs5995653	Modified by ICS and associated with protective response to exacerbations

The stress-induced phosphoprotein 1 (STIP1) modulates chaperone activities of HSP70 and HSP90, which can influence lung function responses to corticosteroids, thus any genetic variation will disturb this functioning (58). *STIP1* is associated with lung function response during ICS therapy (71). On the contrary, research revealed that asthmatic adult individuals who were carriers of the variant form of the (H33Q) nonsynonymous TBX21 rs2240017 polymorphism, had worse control of symptoms when treated with ICSs for 12 weeks, thus suggesting that the mentioned polymorphism is related to response to ICS (72).

In a multi-ancestry population Study of Asthma Phenotypes and Pharmacogenomic Interactions by Race-ethnicity (SAPPHIRE), response to a 6-week ICS was found to impact the rs3827907 variant, which highly correlated with the RNASE2 gene (73). This variant was replicated in GALA 1 and SAGE I populations with Latino and African American populations. RNASE2 is highly expressed in eosinophil granules and can be used as a predictive marker for ICS response, particularly in African American or Latino populations. In another ICS response study in admixed children from GALAII and SAGE cohorts with asthma, rs5995653 SNP related to apolipoprotein B mRNA-editing catalytic polypeptide 3 (APOBEC3)B and APOBEC3C genes was associated with a protective effect against asthma exacerbations (74). This is likely due to the APOBEC3's role in antiviral response and viral exacerbation-induced asthma.

## Short-acting β_2_-agonists

A GWAS study of 949 African American children with asthma under Study of African Americans, Asthma, Genes and Environments (SAGE I and II) receiving albuterol, found a better response to bronchodilators compared to European ancestry children with asthma (75). A smaller cross-sectional study found significantly lower responsiveness in African Americans compared to European Americans at higher doses of albuterol (360 mcg and 540 mcg), suggestive of a dose-dependent response (76). The variant (rs73650726) responsible for differential albuterol response is more common in African Americans (8%) and less frequent in Latino populations (1%) and absent in European and Asian populations (75). Other bronchodilator response genes such as SPATA13-AS1 have been identified using SABA in otherwise healthy 328 African Americans (Table 1). This gene was independently verified in a replication cohort of the Study of Asthma Phenotypes and Pharmacogenomic Interactions by Race-ethnicity (SAPPHIRE) of African Americans and European Americans with asthma (69). It is interesting that no major safety issues have been reported in patients of African ancestry receiving SABA compared to other ethnicities.

## Long-acting β_2_-agonists

Clinical outcomes from multiple randomised controlled trials (RCT) and cohort studies on LABAs responses across different ethnicities are inconclusive (77). Several Cochrane reviews of RCTs have found seriously increased risk of disease deterioration in African Americans associated with LABA therapy compared to other population groups (78–81). African Americans were found to be at high risk of respiratory-related death and hospitalisation requiring intubation and mechanical ventilation, however, LABA therapy did not lead to significantly more asthma-related deaths (80). Some of the increased risk of asthma-related death in African American population can be attributable to treatment failure in patients receiving LABA alone or in combination with other biologics such as Leukotriene receptor agonists (82). Some RCT studies have reported no significant increased risk in African Americans compared to others receiving ICS (fluticasone) alone or in combination with salmeterol in adolescents and adults (83). This was also true for patients of African ancestry receiving a combination of formoterol with budesonide or budesonide alone. Generally, there is a suggestion that people of African ancestry are at higher risk of being hospitalised due to asthma-related symptoms and that some LABA (in this case salmeterol) at certain doses can increase this risk further. More powered observational studies would be essential in determining asthma-related or LABA therapy-related risk amongst patients of African ancestry in order to control asthma more effectively.

## Genetic variants in the β_2_-adrenergic receptor gene

The β_2_-adrenergic receptor gene (ADRB2), a receptor target for beta-agonist therapy has been vigorously investigated (Table 1) (67, 75, 77, 84, 85). ADRB2 is intronless, yet a polymorphic gene with more than 49 different genetic variants in multi-ethnic asthma cohorts evaluated to date (86–88). Of these, the most intensively studied are mutations leading to amino acid changes in position 16 leading to 2 variants (Gly16Gly and Gly16Arg, rs1042713) and changes in position 27 leading to 3 variants (Gln27Glu, Glu27Gln, Gln27Gln, rs1042714) (85). These changes lead to the downregulation of the β_2_-adrenergic receptor (β_2_AR) and resistance to β_2_-adrenergic agonist-induced relaxation of the smooth muscle (89). A meta-analysis study found the ADRB2 genotype (Arg16Arg) to be beneficial in children of African ancestry treated with SABA compared to other ethnicities (89). However, in a LARGE RCT study, the Arg16Arg variant had the opposite effect in the participants of African ancestry treated with LABA together with ICS or LABA alone (82). The Gln27Glu variant has been investigated in both adults and children. An RCT study of 87 patients with the 27Gln variant receiving LABA, showed an age-dependent response, with older patients responding better than younger patients. Younger patients with 27Gln or 27Glu responded better to LABA plus ICS (90). In another pharmacogenetic study of rare *ADRB2* variants, the rare Thr164Ile variant (rs1800888) was associated with asthma-related hospitalization in the past year in non-Hispanic white population and African American asthmatics treated with LABA, respectively (88). Interestingly, the same genotype was associated with fewer hospital visits in non-Hispanic white population not receiving LABA (91).

Adenylyl cyclase type 9 is an enzyme within the canonical β_2_-adrenergic receptor pathway encoded by *ADCY9* (Table 1). ADCY9 coding SNP, Met772Ile (rs2230739), is associated with bronchodilator response to a SABA, albuterol, only in patients treated with an ICS from the Childhood Asthma Management Program (CAMP) cohort (92). This gene pathway interaction was replicated in an independent Korean population trial cohort treated with a LABA, formoterol, in combination with an ICS. In addition, rs1042713 SNP, which conditions the G16R genotype, influences long-term response to SABA, and salbutamol, and patients who are homozygous (16R/R) could benefit from avoiding salbutamol (93).

Other biologics which we have not covered in detail in this review include leukotriene modifiers. The response to treatment with leukotriene modifiers among asthmatics is highly heterogeneous (94). The role of genetics in LTRA still lacks strong evidence. Current data suggest that variants in ALOX5 (rs2115819) (95) and LTC4S (rs730012) likely modulate a portion of the variability in responses to LRTA (59, 96, 97). Robust research as well as replication pharmacogenetics studies in asthmatic patients are still lacking, particularly in patients of African ancestry.

## Future direction

There is a paucity of data on the immunogenetics of asthma in Africa. In this review, we have heavily relied on African American data. We are cognizant of the fact that African American population is composed of a complex admixed group. This is further complicated by the fact that pharmacogenomics of asthma would be largely influenced by the environment, meaning such data cannot be extrapolated to Africa. Studies in this area will give direction in more precise treatment for individual asthmatic patients. Although factors such as access to drugs or adherence may affect symptom control in asthmatic patients in Africa, there may be a subset of patients who do not respond to certain asthma drugs. Differences in minor allele frequencies at drug pharmacogenetic loci potentially contribute to a greater frequency of treatment failures or adverse responses and these need to be considered at the population level and individual level, especially when dealing with recently admixed populations. Currently, no asthma GWAS case-control study or GWAS-based pharmacogenetics study has been performed in Africa which is worrying considering how certain drugs could potentially exacerbate asthma-related complications. There is a need to train computational biologists and bioinformaticians to close a skills shortage gap in Africa which will be able to tackle big data being generated in various consortiums including efforts led by H3Africa. Ethical considerations and policies to guide against the exploitation of vulnerable communities and sensitiveness around unique social, cultural, economic, and religious contexts in Africa need to be at the forefront when considering genomic research to build trust. Genomics research in Africa will require huge investment by global funding bodies and commitment from local governments to ensure sustainability. Bridging the genomics gap in African populations has huge potential for genomic medicine worldwide and importantly will reduce health disparities. The recently established health monitoring database in South Africa will likely combine different omics such as transcriptomics, proteomics, epigenetics, and imaging technologies together with genomics and health electronic records will improve health outcomes and prevent unnecessary morbidity and mortality. In asthma, there is an urgent need for the discovery of GWAS studies based on continental African cohorts and also pharmacogenetics of currently available first-line asthma drugs to lessen the asthma burden in Africa.

## Conclusion

Asthma is a complex disease of high prevalence in Africa. Environmental factors including genetics have been shown to be associated with asthma. Furthermore, several susceptible genetics associated with asthma have been identified in patients of varying ethnicity, however, little information exists on patients of African ancestry. Management of asthma includes ICS and β_2_-adrenergic receptor agonists, although not all patients are controlled by this therapy. Therefore, the identification of gene polymorphism or gene response to therapy is of utmost importance in symptom control and in developing more personalised treatment options.
